# The Reproduction Rate of Peptide Transporter PEPT-1 Deficient *C. elegans* Is Dependent on Dietary Glutamate Supply

**DOI:** 10.3389/fmolb.2018.00109

**Published:** 2018-11-30

**Authors:** Britta Spanier, Jacqueline Wallwitz, Despoina Zapoglou, Bio Maria Ghéo Idrissou, Christine Fischer, Martina Troll, Katrin Petzold, Hannelore Daniel

**Affiliations:** Nutritional Physiology, Technische Universität München, Munich, Germany

**Keywords:** nutrient transporter, amino acid homeostasis, peptides, nematode, intestine, fertility, epithelium

## Abstract

Intestinal absorption of dietary amino acids is mediated via two routes. Free amino acids released by hydrolysis of dietary proteins are taken up by a multitude of amino acid transporters while di- and tripeptides released are taken up by the peptide transporter PEPT-1. Loss of PEPT-1 impairs growth, post-embryonic development and reproduction in *Caenorhabditis elegans*, and supplementation with a mixture of all L-amino acids only partially rescues fertility. In the present study, we demonstrate that dietary L-glutamate is the responsible amino acid that can increase fertility in hermaphrodite *pept-1* worms. This effect was associated with a significantly higher uptake of glutamate/aspartate in *pept-1* than in wildtype *C. elegans*. Furthermore, we found that the intestinal transporter proteins SNF-5 of the solute carrier SLC6 family of nutrient amino acid transporters (NAT) and AAT-6 of the SLC7 family as the light subunit of a heteromeric amino acid transporter (HAT) play a key role in glutamate homeostasis in *pept-1 C. elegans*. Genes encoding these transporters are highly expressed and upon silencing a 95% reduced fertility (*snf-5*) and sterility (*aat-6*) was observed. A subsequent L-glutamate supplementation failed to rescue these phenotypes. Dietary glutamate supplementation did neither influence the feeding frequency, nor did it improve mating efficiency of *pept-1* males. Most strikingly, *pept-1* were more prone to habituation to repeated gentle touch stimuli than wildtype *C. elegans*, and dietary glutamate supply was sufficient to alter this behavioral output by restoring the mechanosensory response to wildtype levels. Taken together, our data demonstrate a key role of L-glutamate in amino acid homeostasis in *C. elegans* lacking the peptide transporter in the intestine and demonstrate its distinct role in reproduction and for neural circuits mediating touch sensitivity.

## Introduction

Reproduction, development, and growth of organisms depend on sufficient nutrient supply via the diet and on efficient uptake systems in intestinal epithelial cells. In *Caenorhabditis elegans*, intestinal transport processes have only been studied for a few subclasses of nutrients as for example for amino acids (Veljkovic et al., 2004; Hagiwara et al., 2012), di- and tripeptides (Meissner et al., 2004), glucose (FGT-1; Feng et al., 2013; Kitaoka et al., 2013), fatty acids (ASC-22, homolog of FATP4 in humans; Kage-Nakadai et al., 2010) or citrate (NAC-2; Fei et al., 2004) and a few others.

For proper growth and reproduction, amino acids, and in particular the supply with essential amino acids, is most critical. Amino acids can be absorbed in peptide-bound form (as di- or tripeptides) or as free amino acids using class-specific transport proteins including heteromeric amino acid transporters (HAT) (Veljkovic et al., 2004), Na^+^-dependent or proton-coupled amino acid transporters (PAT) and exchanger systems (Metzler et al., 2013). Uptake in peptide-bound form is mediated by PEPT-1 belonging to the solute carrier (SLC) family 15. It transports almost all possible di- and tripeptides (up to 400 di- and 8,000 different tripeptides) but also structurally related drugs (ß-lactam antibiotics, selected ACE inhibitors) in a pH-dependent manner (for review see Rubio-Aliaga and Daniel, 2008). PEPT-1 deficiency induces a severe phenotype in *C. elegans*, characterized by 40% reduced body size, 100% prolonged post-embryonic development and 60% reduced reproduction rate when compared to wildtype controls (Meissner et al., 2004), which indicates its central role in amino acid supply in the nematode. In *pept-1* the content of almost all free amino acids is significantly lower than in wildtype *C. elegans*, and this overall amino acid deficiency affects target of rapamycin (TOR) mediated signaling and the *de novo* protein synthesis rate (Geillinger et al., 2014). The associated low reproductive rate in *pept-1* was shown to be partially rescued by supplementation with free amino acids (Meissner et al., 2004). Based on this finding we aimed to identify which amino acid(s) support this improvement in *pept-1* reproduction and which amino acid transporter(s) are most crucial for the absorption of these nutrients from the diet in the *C. elegans* intestine.

## Materials and methods

### *C. elegans* strains and nematode culture

The following *C. elegans* strains were used: wildtype N2 (var. Bristol), *pept-1*(*lg601*), *rrf-3*(*pk1426*), *fem-1(hc17);unc-76(e911)*, and *fog-2(q71)*. The last two strains were obtained from Eric Lambie and Barbara Conradt (Munich). Strains were grown and maintained at 20°C as a mixed population which contains all developmental stages (eggs, L1-L4 larvae, adults) on nematode growth medium (NGM) agar plates seeded with *E. coli* OP50 (Stiernagle, 2006). The *fog-2 C. elegans* were maintained as a male/female population. For RNA interference (RNAi) experiments *C. elegans* were fed for at least 3 days (development from L1 to L4 larvae) with the corresponding *E. coli* HT115 clones from the Ahringer *C. elegans* RNAi library following the protocol described earlier (Fraser et al., 2000). As control, *C. elegans* were grown on *E. coli* HT115 containing only the empty vector pPD129.36 (L4440) (hereafter called vector control, vc).

### Reproduction rate

To analyze the impact of amino acid supplementation on reproduction, synchronized *pept-1* L1 larvae were fed on *E. coli* OP50 bacteria supplemented on top with an amino acid mixture containing 12 amino acids (0.9 mM L-arginine, 0.28 mM L-aspartate, 0.24 mM L-asparagine, 0.32 mM L-histidine, 0.31 mM L-cysteine, 0.26 mM L-glutamate, 0.13 mM L-methionine, 0.27 mM L-phenylalanine, 0.24 mM L-serine, 0.47 mM L-threonine, 0.06 mM L-tryptophan-, 0.38 mM L-tyrosine, final concentrations in agar) [given concentrations are equal to a 1:1 mixture of MEM (minimum essential medium) non-essential amino acids (100x) and MEM amino acids (50x) without L-glutamine (Sigma-Aldrich, Taufkirchen, Germany)], with a single amino acid (0.32 mM L-histidine, 0.31 mM L-cysteine,0.26 mM L-glutamate, or 0.26 mM L-glutamine), or with a mixture of all amino acids except for L-glutamate and L-glutamine until they reached the L4 larval stage. The L4 larvae from each supplementation group were then singled on freshly prepared plates containing the corresponding supplementation and developed into adults under the same conditions they had during their post-embryonic development. The offspring of each individual animal was counted.

The role of selected amino acid transporters in glutamate supply was analyzed using an RNAi approach. *pept-1* L1 larvae were fed on *E. coli* HT115 expressing dsRNA for the *snf-5* or the *aat-6* gene until they reached the L4 larval stage. The L4 larvae were cultured individually on agar plates seeded with *E. coli* HT115 expressing *snf-5* or *aat-6* dsRNA with and without 0.26 mM L-glutamate. The worms developed into adults and the offspring of each worm was recorded.

### Pharyngeal pumping assay

*pept-1* L1 larvae were grown on NGM agar plates with *E. coli* OP50 and supplemented with 0.26 mM L-glutamate, 0.26 mM D-glutamate, 0.26 mM L-glutamine, or water (control) until they reached the L4 larval stage. Individual L4-larvae were transferred to freshly prepared NGM agar plates with *E. coli* OP50 and supplemented with 0.26 mM L-glutamate, 0.26 mM D-glutamate, 0.26 mM L-glutamine or water (control). After 5 min the number of pharyngeal pumps within 1 min was counted for each animal.

### Behavioral assay for touch sensitivity

The response to a gentle touch stimulus of wildtype N2 and *pept-1 C. elegans*, with and without lifelong L-glutamate supplementation, was quantitatively assayed. N2 and *pept-1* eggs were grown on NGM agar plates with *E. coli* OP50 supplemented with 0.26 mM L-glutamate or water (control) until they reached the young adult stage (adults with 2–4 embryos in their uterus). Freely moving individual worms were selected for the assay. The stimulus was delivered by a human eyelash as alternating strokes to the head (behind the pharynx) and the tail (after the anus and before the tail tip). Each stroke counted as one and the total number of strokes that elicited forward or backward locomotory response was scored. Tested animals were killed to avoid examining the same animal twice.

### Mating efficiency of *pept-1* males

Mating assays were performed to quantitatively investigate the impact of L-glutamate intake throughout male *pept-1 C. elegans* development on its fertility by scoring the number of progeny of individual males. After development from egg to adult on NGM agar plates with *E. coli* OP50 supplemented with 0,26 mM L-glutamate, 1 day adult *pept-1* males were individually mated with a *pept-1(lg601)* hermaphrodite, a *fog-2(q71)* female, or a *fem-1(hc17ts);unc-76(e911)* feminized hermaphrodite which had not been in contact with L-glutamate. *fem-1(hc17ts);unc-76(e911)* feminized hermaphrodites were obtained by maintaining *fem-1;unc-76* eggs at 25°C during development. Each mating pair was placed on 3.5 cm NGM agar plates with *E. coli* OP50 as food source but without L-glutamate supplementation. The nematodes were permitted to interact exhaustively with each other in all assays and the total number of progeny for each mating pair was scored.

### LC-MS/MS based analysis of amino acid profiles

Mixed populations, containing all developmental stages, of *ad libitum* fed wildtype and *pept-1 C. elegans* grown on 9 cm NGM-agar plates were harvested and washed in M9 buffer. For each strain worms from at least five plates were collected for one sample and in total three biological replicates were prepared per strain. Fifty microliter pellet samples were incubated in reaction tubes with 200 μl complex amino acids mixture [1:1 of MEM (minimum essential medium) non-essential amino acids (100x) and MEM amino acids (50x) without L-glutamine (Sigma-Aldrich, Taufkirchen, Germany)] in presence of *E. coli* OP50. As controls *E. coli* OP50 were incubated with the complex amino acid mixture, and 50 μl *C. elegans* wildtype and *pept-1* pellet samples were incubated in water. Medium samples were collected at time points 0, 60, 180, and 360 min. The worm pellets were collected at 360 min, washed twice with M9 buffer and once with distilled H_2_O, and were snap frozen in liquid nitrogen. The protein content of each sample was analyzed by the Bradford Protein Assay. Acetonitrile (LC-MS grade), ammonium acetate, formic acid (LC-MS grade), phenyl isothiocyanate (PITC), and pyridine were purchased from Sigma-Aldrich (Taufkirchen, Germany). LC-MS grade water was purchased from J. T. Baker Chemicals (Center Valley, PA). Ethanol and methanol (both LC-MS grade) were obtained from Merck (Darmstadt, Germany). The internal standard (IS) Masschrom from ChromSystems (München, Germany) was used and expanded by glutamine-D5 and asparagine-15N2 (20.0 μmol/l each), both from Cambridge Isotope Laboratories, Inc. (Andover, USA), and tryptophan-D5 (2.0 μmol/l) from Santa Cruz Biotechnology, Inc. (Dallas, USA). Analytes for the external standard (ES) solution comprised the amino acids glycine, L-alanine, L-arginine, L-asparagine, L-aspartic acid, L-citrulline, L-glutamic acid, L-glutamine, L-histidine, L-isoleucine, L-leucine, L-lysine, L-methionine, L-ornithine, L-phenylalanine, L-proline, L-serine, L-threonine, L-tryptophan, L-tyrosine, and L-valine purchased from Sigma-Aldrich (Taufkirchen, Germany). The total content of free amino acids in the medium samples was determined on a triple quadrupole 3200 Q Trap LC-MS/MS system (AB Sciex, Framingham, MA) with a 1200 Series binary pump, a degasser, and a column oven (Agilent, Santa Clara, CA). To increase the sensitivity and specificity of the amino acid analysis pre-column derivatization using phenyl isothiocyanate (PITC) was performed as described by Cohen and Strydom (1988). 10 μl of each sample and 25 μl internal standard were dried under a stream of nitrogen for 20 min prior to incubation with 190 μl of the derivatization reagent (1:1:1 Pyridin/EtOH/0.1% NH_3_) with shaking at 25°C and 750 rpm for 5 min 10 μl of PITC were added, samples were shaken at 25°C and 750 rpm for 20 min and subsequently dried under nitrogen flow for ~60 min. For metabolite extraction, 100 μl of 5 mM ammonium acetate in methanol were added and samples were shaken at 25°C and 750 rpm for 10 min 70 μl of each sample were diluted with 30 μl LC-MS water and the microplate was applied to the autosampler for measurement. For calibration, control plasma (ClinChek control plasma (human) for amino acids, Recipe, Munich, Germany) with varying amino acid concentrations as well as external standard containing all amino acids added in increasing amounts, were measured in parallel as separate samples. The parameters of the ion spray source operating in positive ESI mode were set to: curtain gas −20 psi, collision gas - medium, ion spray voltage −5,500 V, temperature −500°C, ion source gas 1 – 40 psi, ion source gas 2 – 50 psi. Chromatographic separation was accomplished by a VDSpher 100 PUR C18-SE column (length 150 mm, internal diameter 3.0 mm, particle size 3.5 μm; VDS optilab), at a column temperature of 50°C. Eluent A was made up of 0.2% formic acid in water, and eluent B consisted of 0.2% formic acid in acetonitrile. Gradient elution comprised the following steps: A linear decrease from 98 to 60% A at 500 μl/min over 6 min, hold for 2 min, followed by a linear decrease to 0% A at 500 μl/min over 7 min. Re-equilibration was achieved by a linear increase to 98% A at 500 μl/min over 1 min, increase of flow rate to 800 μl/min over 1 min, hold for 2 min, and decrease to 500 μl/min over 1 min, leading to a total running time of 20 min. Analytes were quantified in scheduled multiple reaction monitoring (MRM) with a target scan time of 1 s and a detection window of 60 s. The quadrupoles were set to unit resolution. Calibration curves, the areas under the curve (AUC) of amino acid concentrations, and data analysis were performed using Analyst 1.5 software (AB Sciex, Darmstadt, Germany). Raw data were corrected for the total protein concentration of the samples as determined by the Bradford Protein Assay (Supplemental Table S1). Additionally, the values of the samples that contained *E. coli* OP50 with the same amino acid solution were subtracted from those of the samples containing *C. elegans*, amino acids and *E. coli* OP50 to eliminate the bacteria-specific changes. The values of the samples that contained *C. elegans* and water instead of amino acids were also subtracted to eliminate metabolites secreted by *C. elegans*.

### Transcriptomics data analysis

Synchronized L4-larvae cultures of wildtype and *pept-1 C. elegans* (*n* = 5) were subjected to transcriptome analysis using *C. elegans* whole-genome DNA microarrays (Affymetrix Inc., Santa Clara, CA, USA). Parts of the data sets have been published before (Spanier et al., 2009, 2010; Martin et al., 2011). The nematode culture, RNA preparation, microarray analysis, data analysis, and statistics were performed according to described protocols (Spanier et al., 2009).

### Statistical analysis

To calculate differences between treatment groups, the Student's *t*-test or one-way ANOVA followed by Tukey's *post-hoc* test was performed. Comparison of groups over time was carried out with mixed model two-way ANOVA with Bonferroni post-test.

## Results

### Glutamate supports reproduction in *pept-1* deficient *C. elegans*

**Figure 1:**
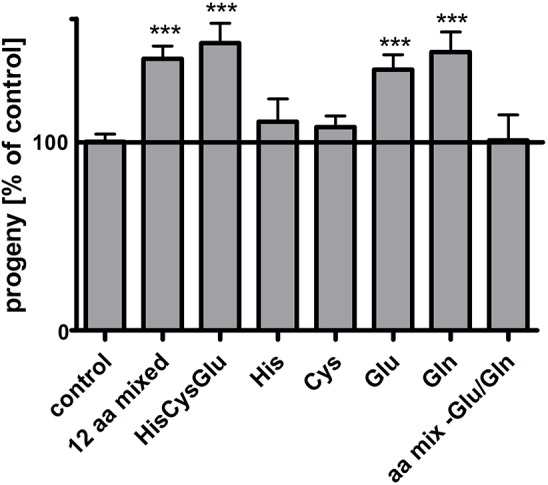
Glutamate improves the reproduction of *pept-1(lg601) C. elegans*. Impact of amino acid supplementation on *pept-1* hermaphrodites. *pept-1* L1 larvae on *E. coli* OP50 were supplemented with an amino acid mixture containing 12 amino acids (0.9 mM L-arginine, 0.28 mM L-aspartate, 0.24 mM L-asparagine, 0.32 mM L-histidine, 0.31 mM L- cysteine, 0.26 mM L-glutamate, 0.13 mM L-methionine, 0.27 mM L-phenylalanine, 0.24 mM L-serine, 0.47 mM L-threonine, 0.06 mM L-tryptophan, 0.38 mM L-tyrosine, final concentrations in agar), with a mixture of 0.32 mM L-histidine, 0.31 mM L-cysteine, 0.26 mM L-glutamate, with a single amino acid (0.32 mM L-histidine, 0.31 mM L-cysteine, 0.26 mM L-glutamate or 0.26 mM L-glutamine), or with a mixture of all amino acid except for L-glutamate and L-glutamine until they reached the L4 larval stage. The offspring of individual animals was counted. Values are presented as mean ± SEM (*n* ≥ 18 individual worms per group; ^***^P < 0.001; Students *t*-test).

It has previously been shown that the reproduction rate of *pept-1 C. elegans* hermaphrodites is doubled after supplementation with a mixture of all L-amino acids except for glutamine, while the amino acids did not change the fertility of wildtype *C. elegans* (Meissner et al., 2004). To identify the amino acid(s) in the mixture exerting this positive effect on *pept-1* reproduction rate, in a first phase we could reduce the number to 12 selected L-amino acids (Arg, Asp, Asn, His, Cys, Glu, Met, Phe, Ser, Thr, Trp, Tyr) (Figure 1). In a second phase a mixture comprising L-histidine, L-cysteine and L-glutamate was still effective and each of them was in turn tested separately. L-histidine and L-cysteine did not lead to any detectable changes, whereas L-glutamate supplementation induced a significant increase in the reproduction rate of *pept-1* hermaphrodites. Supplementation with L-glutamine, which is quickly deamidated in metabolism to L-glutamate by glutaminase, had the same effect. A reconstitution of the initial mixture of amino acids but lacking glutamine and glutamate failed to affect reproduction, leaving L-glutamate as the most critical amino acid in *pept-1* hermaphrodite fertility.

### Dietary glutamate absorption is high in *pept-1 C. elegans*

**Figure 2:**
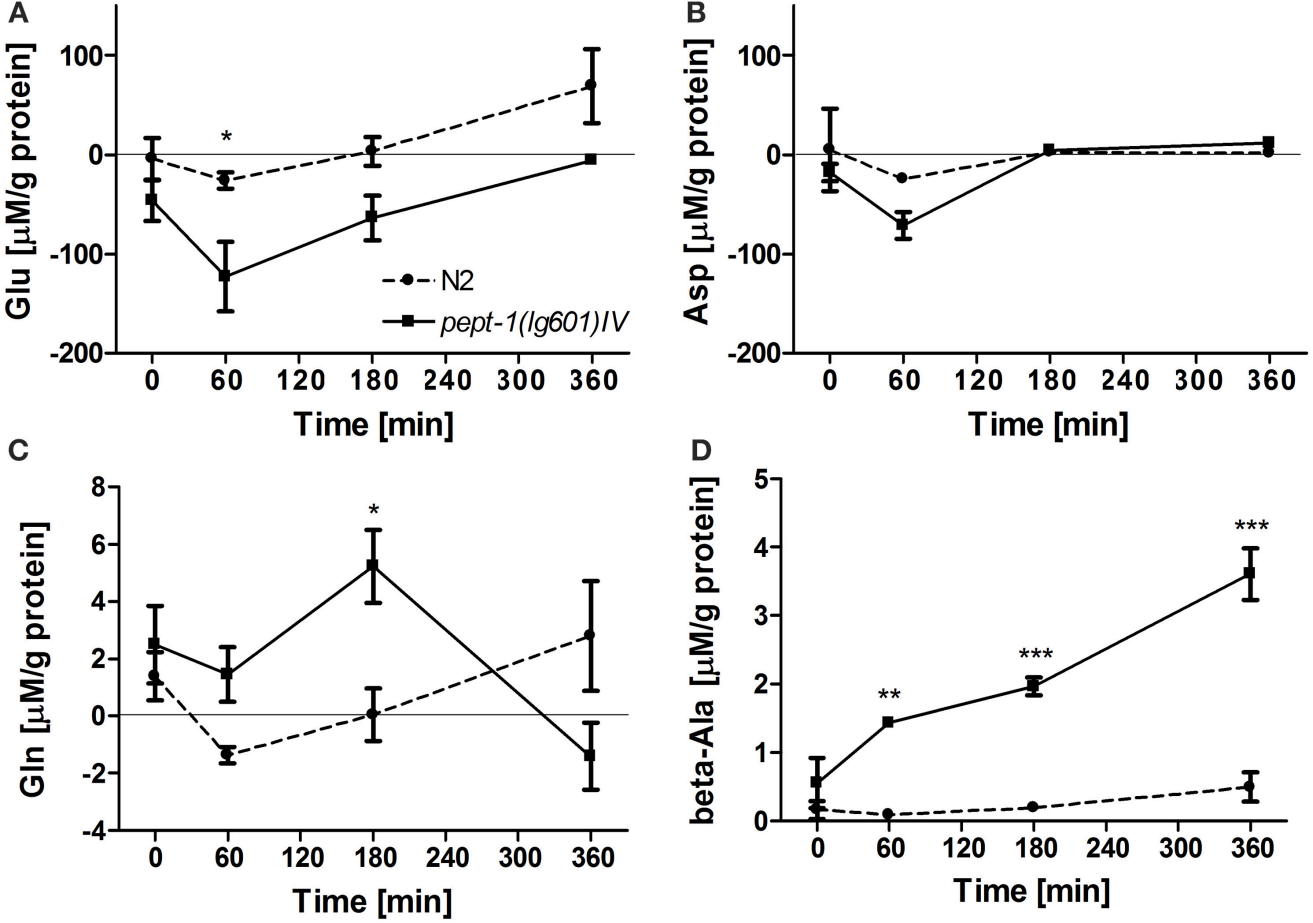
Amino acids with concentration changes over time in the culture medium of wildtype (N2) and *pept-1(lg601) C. elegans*: L-glutamate (Glu) **(A)**, L-aspartate (Asp) **(B)**, L-glutamine (Gln) **(C)**, and ß-alanine (beta-Ala) **(D)**. Samples were analyzed after 0, 60, 180, and 360 min. The values of the samples that contained *E. coli* OP50 with the same amino acid solution were subtracted from those of the samples containing *C. elegans*, amino acids and *E. coli* OP50 to eliminate the bacteria-specific changes. The values of the samples that contained *C. elegans* and water instead of amino acids were also subtracted to eliminate metabolites secreted by *C. elegans*. Values are presented as mean ± SEM (*n* = 3 biological replicates; ^*^P < 0.05, ^**^P < 0.01, ^***^P < 0.001; mixed model two-way ANOVA with Bonferroni post-test).

We used a targeted metabolomics approach to analyze amino acid patterns in *pept-1* deficient *C. elegans* compared to wildtype animals. The concentration of glutamate in the culture medium of *pept-1 C. elegans* was significantly lower after incubation than that in the culture medium of wildtype with most pronounced affects observed after 60 min of incubation (Figure 2A). The concentration of aspartate dropped within the same period in a similar manner (Figure 2B), indicating that the higher absorption rate of these two anionic amino acids is mediated by the same intestinal transport protein as it is found in mammals (Bailey et al., 2011). On the other hand, *pept-1 C. elegans* were shown to secrete glutamine and beta-alanine into the medium reaching up to 5- and 3.5-fold higher levels than in the medium of wildtype control animals (Figures 2C,D). These secretion rates however were, compared to the glutamate and aspartate absorption rates, around 10-fold lower. Moreover, the secretion of glutamine declined after 180 min of incubation, reflecting its absorption by *pept-1 C. elegans*—as indicated by the much lower concentration of this amino acid in the medium at 360 min. A comparable pattern with secretion at 60 and 180 min and absorption at 360 min was also found for alanine. The concentrations of proline, methionine, and phenylalanine were significantly decreased between 180 and 360 min and a similar reduction was observed for arginine although it was not significant (Supplemental Figure S1, Supplemental Table S1). Bacteria-specific changes can be excluded because they were controlled for during the qualitative analysis. Other tested amino acids did not show significant changes in their medium concentration including the three branched-chain amino acids (BCAA) leucine, isoleucine and valine. These findings might be explained by a potentially very low secretion of essential amino acids by *C. elegans*. Assuming that a high uptake of glutamate supports BCAA biosynthesis *in vivo*, we would not expect to find these BCAAs in the culture medium.

### SNF-5 and AAT-6 are important for glutamate transport in *pept-1 C. elegans*

**Figure 3:**
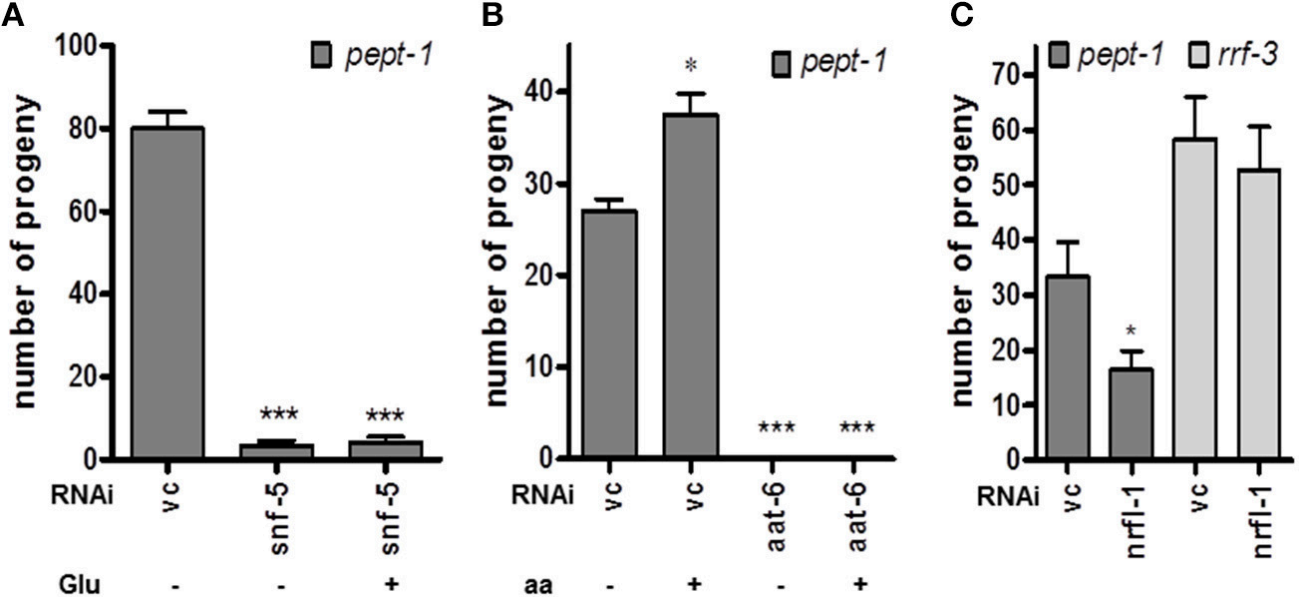
Impact of selected amino acid transporters on the reproduction of *pept-1(lg601) C. elegans*. **(A)** RNAi gene silencing of the cysteine/glutamate transporter *snf-5* reduced the number of progeny compared to control (vc) independently of L-glutamate supplementation (*n* ≥ 19 individual worms per group; ^***^P < 0.001; Student's *t*-test. **(B)** Supplementation with a mixture of essential and non-essential amino acids (aa) had a positive impact on reproduction in *pept-1 C. elegans* with vc RNAi but not in worms with *aat-6* gene silencing (*n* ≥ 31 individual worms per group; ^*^P < 0.05, ^***^P < 0.001; Student's *t*-test). **(C)** Gene silencing of *nrfl-1*, which codes for a scaffold protein of AAT-6, significantly reduced the progeny number of *pept-1* but not of *rrf-3 C. elegans* (*n* ≥ 29 individual worms per group; ^*^P < 0.05; Student's *t*-test). Values are presented as mean ± SEM.

We used our mRNA expression data set to identify differences in expression levels for relevant amino acid transporters, and that revealed a 4.4-fold higher mRNA level of the *snf-5* gene in *pept-1 C. elegans* compared to wildtype controls. *snf-5* codes for a cysteine/glutamate transporter that is expressed in the intestine, selected neurons and the rectal gland cell (Metzler et al., 2013) and shows homology to mammalian and insect nutrient amino acid transporters (NATs). Moreover, the *aat-6* mRNA, which most probably codes for one of the nine HAT light subunit homologous (*aat-1*–*aat-9*) (Hagiwara et al., 2012), was 1.5-fold higher expressed in *pept-1 C. elegans*. Silencing of these genes in *pept-1* deficient *C. elegans* lead to an around 95% reduced fertility in case of *snf-5* and to sterility in case of *aat-6* (Figures 3A,B). Glutamate (Figure 3A) or a mixture containing all L-amino acids (Figure 3B) was not able to rescue these phenotypic changes, although in *pept-1* (vc RNAi) *C. elegans* the supplementation did. This suggests an important physiological role of these two transport proteins in amino acid homeostasis in *C. elegans* when the intestinal peptide transporter PEPT-1 is inactive. Furthermore, silencing the gene coding for the scaffolding protein NRFL-1, which was shown to anchor AAT-6 in the plasma membrane (Hagiwara et al., 2012), also lead to a 50% reduced fertility in *pept-1 C. elegans*. The effect was absent in the RNAi-sensitive strain *rrf-3*(*pk1426*) that expresses a functional peptide transporter PEPT-1 (Figure 3C). These findings together indicate a central role of SNF-5 and AAT-6 in glutamate absorption and overall amino acid homeostasis to support reproduction in *pept-1* deficient *C. elegans*.

### Phenotypic modification induced by glutamate supply in *pept-1 C. elegans*

**Figure 4:**
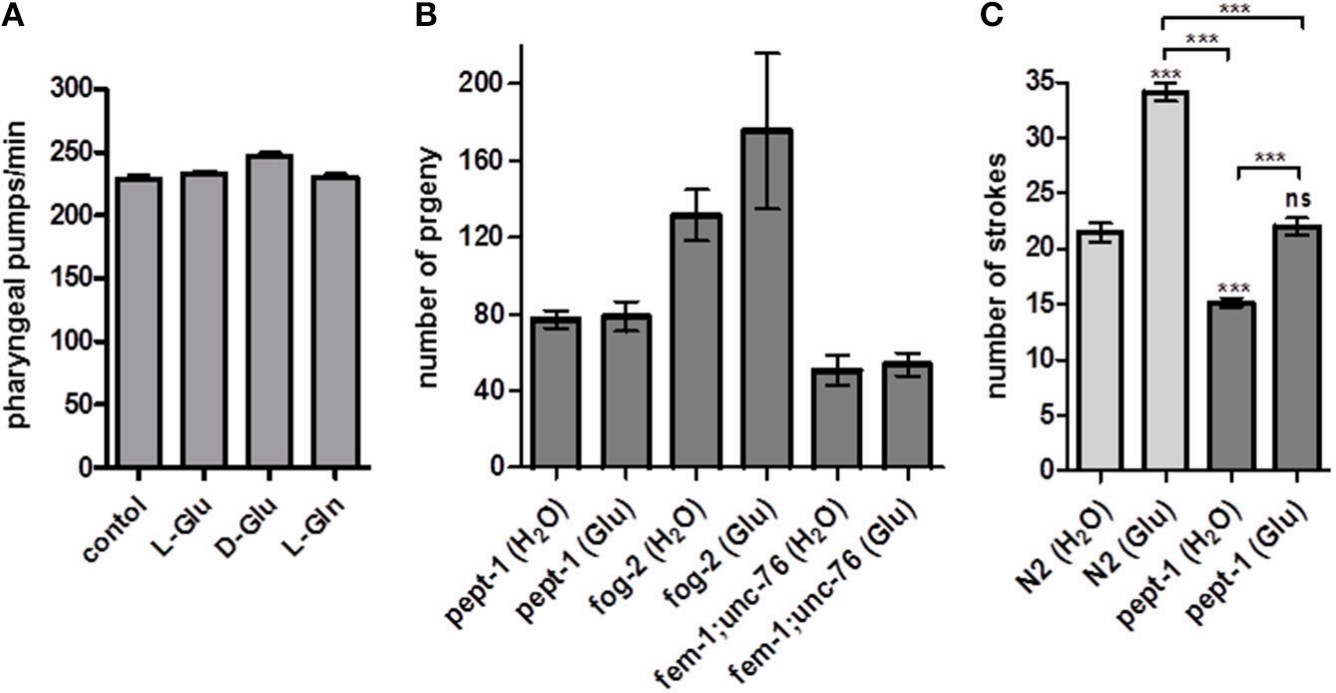
Effect of L-glutamate on feeding behavior, male mating efficiency and response to gentle touch in *pept-1 C. elegans*. **(A)** Pharyngeal pumping frequency of *pept-1 C. elegans* on *E. coli* OP50 supplemented with L-glutamate, D-glutamate and L-glutamine (*n* ≥ 8 individual worms per group). **(B)** Mating efficiency of *pept-1* males with *pept-1* hermaphrodites (*n* ≥ 20), *fog-2(q71)* females (*n* ≥ 7), or *fem-1(hc17ts);unc-76(e911)* feminized hermaphrodites (*n* ≥ 12) with and without L-glutamate supplementation. n indicates the number of mating pairs. **(C)** Mechanosensory response of wildtype and *pept-1 C. elegans* with and without L-glutamate supplementation (*n* ≥ 19 individual worms per group; ^***^P < 0.001; ANOVA with Tukey's multiple comparison test). Values are presented as mean ± SEM.

Glutamate is known as a neurotransmitter and modulator of *C. elegans* behavior. The pharyngeal contraction and pumping behavior is triggered by glutamate via the metabotropic glutamate receptor MGL-1 (Dillon et al., 2015). To exclude an altered food and nutrient uptake in *pept-1 C. elegans* when supplied with external free glutamate, we measured pharyngeal pumping frequency under *ad libitum* bacterial food supply as standard condition. No significant changes were observed in the pumping rate of *pept-1 C. elegans* exposed to free L-glutamate, D-glutamate or L-glutamine (Figure 4A), excluding the possibility that differences in food intake account for the effects on reproduction.

The question therefore remains: how does extra glutamate affect fertility and/or reproduction? Since the number of offspring in *C. elegans* is determined by the number of available sperm and not by the oocytes (Ward and Carrel, 1979), we hypothesized that glutamate might influence sperm cells. It has recently been shown that free glutamate has a positive effect on pig sperm-oocyte interaction during *in vitro* fertilization (Spinaci et al., 2017). In humans and mice, sperm capacitation is promoted by the fertilization promoting peptide pGlu-Glu-Pro-NH_2_, a glutamate-containing tripeptide structurally similar to thyrotropin releasing hormone (TRH) (Fraser et al., 2006). To exclude a possible effect of glutamate on oocytes, the mating efficiency of *pept-1* males was tested. For that purpose *pept-1* males that developed in the absence or the presence of extra glutamate were mated in a 1:1 ratio with a *pept-1(lg601)* hermaphrodite, a *fog-2(q71)* female, or a *fem-1(hc17ts);unc-76(e911)* feminized hermaphrodite. Although, the mating efficiency was influenced by the female/feminized hermaphrodite genotype, glutamate supplementation had no significant effect on the total number of progeny (Figure 4B). Wildtype males supplemented with glutamate or water paired with *fem-1(hc17ts)IV;unc-76(e911*) sired 51 ± 12 and 46 ± 16 progeny, respectively (mean ± SD), and when paired with *fog-2(z71)* female worms they generated 192 ± 122 and 181 ± 72 progeny, respectively (data not shown). Based on these findings we concluded that the positive effect of glutamate on *pept-1* fertility cannot, *per se*, be explained by a positive influence on the sperm cells.

Glutamate is also known to modulate mechanosensory responses in *C. elegans*. For example, the body touch neurons AVM, ALM, PLM, PVM, responsible for the sensation of gentle touch stimuli, are glutamatergic (Lee et al., 1999). We thus tested whether touch sensitivity is altered by *pept-1* deficiency and whether glutamate supplementation could influence neuronal excitation caused by gentle touch stimuli. The response of *C. elegans* to gentle touch decreases when the stimulus is repeated; this process is termed habituation (Chalfie and Sulston, 1981). We found that wildtype and *pept-1 C. elegans* which were supplemented with glutamate throughout their post-embryonic development had a significantly increased response to gentle mechanical stimulation and needed 1.5-fold more strokes until habituation than the control group (Figure 4C). However, this effect was independent of the genotype. More importantly, the response of *pept-1 C. elegans* (15 ± 2 strokes (without Glu), 22 ± 3 strokes (with Glu), mean ± SD) was significantly less intense than that of the wildtype controls [22 ± 4 strokes (without Glu), 34 ± 4 strokes (with Glu)]. This abnormal mechanosensory response might be best explained by the 40% decreased pool of free glutamate in *pept-1 C. elegans* (Geillinger et al., 2014). That may also lead to reduced levels of free glutamate in neuronal cells leading in turn to a faster desensitization of the neuronal circuit. When we supplied free glutamate by supplementation it rescued the phenotype in *pept-1 C. elegans* and restored the mechanosensory response to wildtype levels.

## Discussion

*C. elegans* lacking the intestinal peptide transporter suffer from an amino acid-deficiency that suppresses growth, post-embryonic development and reproduction. These severe phenotypic changes occur because the loss of the intestinal di- and tripeptide transport is only partially compensated by uptake of free amino acid via the different amino acid transporters present in the intestinal epithelium. However, when the bacterial food is enriched with all L-amino acids (except for glutamine because of its instability), the fertility of *pept-1* hermaphrodites is increased (Meissner et al., 2004). In the present study, we found L-glutamate to be the prominent effector amino acid of this process. Moreover, we demonstrated a significantly higher absorption rate of L-glutamate from the culture medium by *pept-1* than wildtype *C. elegans* which is most probably mediated by the transport proteins SNF-5 and AAT-6. Both corresponding genes *snf-5* and *aat-6* have a higher mRNA expression level in animals lacking the peptide transporter, indicating their compensatory role in amino acid homeostasis. Both transporters seem to be crucial for the uptake of L-glutamate to promote embryo development and reproduction. SNF-5 belongs to the SLC6 family with the highest similarity to mammalian and insect NATs and transports, next to numerous neutral amino acids, the acidic amino acids glutamate and aspartate (Metzler et al., 2013). The transport is Na^+^- and K^+^-dependent and specific for L-enantiomers. The property to transport acidic amino acids is unique for a member of the SLC6 transporter family because those transporters usually belong to the SLC1 family. In *C. elegans*, the SLC1 family is represented by the glutamate transporters GLT1-7 but no corresponding gene showed mRNA expression changes in *pept-1 C. elegans* (data not shown). The amino acid transporter AAT-6 is structurally homologous to the light subunit of the HATs class. HATs are composed of a catalytic multi-transmembrane spanning subunit (light subunit, SLC7 family) and a type-II N-glycoprotein subunit (heavy chain, SLC3 family) linked by a disulfide bond. Of the nine amino acid transporter (AAT) homologs in *C. elegans*, AAT-1, AAT-2, and AAT-3 have the highest sequence similarities to the HAT light chains in family SLC7A5. They bind to the glycoprotein heavy chain ATGP-2 via a disulfide bond to form functional heteromeric AATs in the heterologous expression system of *Xenopus* oocytes (Veljkovic et al., 2004). However, gene silencing experiments of all possible candidates known to interact with each other to form functional heterodimers (AAT-1, AAT-3 and ATGP-1, ATGP-2) either individually or in duplicates (AAT-1/ATGP-2 and AAT-3/ATGP-2) had no impact on reproduction of *pept-1 C. elegans* (data not shown). AAT-4 to AAT-9 have the lowest sequence similarities to mammalian HATs and, due to lack of a cysteine residue, cannot interact with the heavy chain proteins ATGP-1 or ATGP-2 (Veljkovic et al., 2004). The interaction of AAT-6 with the scaffold protein sodium-proton exchanger regulatory factor NRFL-1 has already been demonstrated (Hagiwara et al., 2012). Its function in amino acid homeostasis and the link to reproduction we showed by a reduction of reproduction of *pept-1 C. elegans* when the *nrfl-1* gene was silenced. However, whether NRFL-1 also anchors other AAT homologs to the membranes of the intestinal and excretory tract remains unknown. In mammals, SLC7A11 (xCT, cysteine/glutamate exchanger, interacts with 4F2hc) and SLC7A13 (AGT1, aspartate/glutamate transporter, interacts with rBAT) are members of the SLC7 family that accepts acidic amino acids as substrates (Burdo et al., 2006; Nagamori et al., 2016). Therefore, a functional role of AAT-6 in *C. elegans* glutamate/aspartate transport is very plausible.

L-Glutamate belongs to the non-essential amino acids in mammals and in *C. elegans* (Vanfleteren, 1980; Braeckman et al., 2009), and its role as neurotransmitter and modulator of behavior has been described in numerous studies (Zheng et al., 1999). Furthermore, its role as taste enhancer by activation of umami taste receptors in mammals is discussed (Ackroff and Sclafani, 2016). In this study, we were able to show that supplementation of bacterial food with L-glutamate, D-glutamate and L-glutamine did not change the feeding behavior of *ad libitum* fed *pept-1 C. elegans*, and thus a generally increased nutrient supply leading to an improved metabolic status in this amino acid deficient strain could be excluded under the given experimental conditions.

To test the effects of glutamate on the sperm cells, which are the limiting factor of *C. elegans* fertility and dictate the number of offspring (Ward and Carrel, 1979), we performed single male mating assays in a 1:1 ratio with female, feminized hermaphrodite or hermaphrodite worms. These single pair conditions and the severe phenotype (amino acid deficiency, smaller body size) of *pept-1* males might be the reason for the relatively low numbers of offspring. In wildtype *C. elegans* mating stimulates oogenesis (Ward and Carrel, 1979) and when males mate with their conspecific hermaphrodites they can sire 50% male progeny and up to 1,400 eggs (Chasnov and Chow, 2002). However, these data are often from experiments with mating groups, where each hermaphrodite successfully copulated with many males and received high sperm numbers. Glutamate supplementation did not alter the mating efficiency of *pept-1* males in any of the tested mating conditions, and therefore seems to have no positive influence on sperm cells in general.

Although, glutamate did not have a direct effect on feeding behavior nor on sperm cells, it may have effects on metabolic processes that in turn support fertility. Amino acid metabolism leaves nitrogen for excretion as it is toxic, and this is usually achieved by eliminating directly ammonia or by detoxification with production of more water-soluble urea or uric acid. In both *C. elegans* and *C. briggsae*, ammonia is an important product of nitrogen excretion (Rothstein, 1963; Adlimoghaddam et al., 2015), but in *C. briggsae*, tracer studies with radioactively labeled substrates (most dominantly seen in the presence of acetate-2-^14^C) have also demonstrated excretion of nitrogen in the form of various amino acids (Rothstein, 1963, 1965), such as of glutamine in presence of ammonia and glutamate. Beta-alanine is the biogenic amine produced from aspartate and appears to be excreted in case of a high aspartate status. We observed a marked increase in both beta-alanine and glutamine secretion by *pept-1* deficient *C. elegans* when they absorbed high amounts of glutamate and aspartate from the culture medium. This secretion however could also be an indication for exchange processes underlying amino acid absorption as some of the membrane transporters for amino acids mediate such exchange of non-essential vs. essential substrates. However, the excreted glutamine and beta-alanine concentrations represented only 10% of the absorbed glutamate and aspartate. The drop in the concentration of glutamine in the medium after 360 min of incubation might be explained by a change in the metabolic status of *pept-1 C. elegans*, which already suffer from amino acid deficiency, in the present experimental conditions. This drop in their concentration in the medium was also seen for five other amino acids, including the essential methionine, phenylalanine and arginine, and further supports the depletion hypothesis.

In mammalian intestinal epithelial cells glutamate is rapidly metabolized and only minor amounts of dietary glutamate and aspartate enter the portal blood (Wu, 1998). The highest amount of glutamate is converted to alpha-ketoglutarate by glutamate-dehydrogenase and enters the TCA cycle for ATP-production. There seems thus no need for an export of glutamate from epithelial cells into blood, and this may also explain why no specific exporter for glutamate in the basolateral membrane of enterocytes has been identified yet. Assuming that these processes are similar in *C. elegans*, glutamate supplementation should increase the metabolic flux through TCA cycle, and elevate ATP-production and energy supply by intestinal epithelial cells. Glutamine could have the same effect as it is converted to glutamate *in vivo* by glutaminase. Previous work has shown that *pept-1 C. elegans* excretes succinate into axenic culture medium CeHR that contains free L-glutamate in mM concentation (Martin et al., 2011), which may reflect a high TCA cycle activity overloading ATP-production capacity with release of intermediates from TCA cycle into media.

However, the behavioral assay for mechanosensation in both wildtype and *pept-1 C. elegans*, demonstrated that dietary L-glutamate does in part reach neurons to influence neurotransmission. Most probably due to a systemic glutamate deficiency, which was shown previously by a metabolomics approach (Geillinger et al., 2014), *pept-1* are more prone to habituation to gentle touch than wildtype *C. elegans*. This phenotype was rescued by glutamate supplementation and the mechanosensory response of *pept-1 C. elegans* was restored to wildtype levels. As this behavior is dependent on glutamatergic neurons (Lee et al., 1999), the reuptake of glutamate from the synaptic cleft and other tissues via GLT glutamate transporters from the SLC1 family might play a central role in this process (Mano et al., 2007). Furthermore, AAT-6 function in the context of glutamate household in *C. elegans* will be a future issue. An involvement of SNF-5 in these processes is unlikely because it is expressed in amphid sensory neurons ASI, ASK, and ADF, which are responsible for chemosensation of food, in particular amino acids (Metzler et al., 2013). In summary, our data indicate that the behavioral assay for gentle touch sensitivity could be employed as a qualitative analysis of the glutamate pool in *C. elegans*.

## Authors contributions

BS, HD conceived and designed the experiments. BI, JW, DZ, CF, MT, KP, and BS performed the experiments. BS, BI, JW, DZ analyzed the data. HD contributed reagents, materials, analysis tools. BS planned and wrote the manuscript.

### Conflict of interest statement

The authors declare that the research was conducted in the absence of any commercial or financial relationships that could be construed as a potential conflict of interest. The handling editor and reviewer AE declared their involvement as co-editors in the Research Topic, and confirm the absence of any other collaboration.
