# Supplementary material for: The Reproduction Rate of Peptide Transporter PEPT-1 Deficient C. elegans Is Dependent on Dietary Glutamate Supply
Source: Front Mol Biosci. 2018 Nov 30;5:109. doi: 10.3389/fmolb.2018.00109 (PMC6284198; doi:10.3389/fmolb.2018.00109)
Supplement: Supplementary file 1 [file Table_1.pdf]

Amino acid concentation in culture medium [µmol/l]

Sample	group	time [min]	medium	Gly	Ala	Ser	Pro	Val	Thr	Ile	Leu	Asn	Asp	Gln	Glu	Met	His	Phe	Arg	Cit	Tyr	Trp	Orn	Lys	bAla
1	WT	0	OP50+AA	725,00	2570,00	198,00	744,00	1490,00	714,00	2260,00	3590,00	2,28	1380,00	23,80	1070,00	587,00	472,00	1080,00	1850,00	20,30	554,00	109,00	62,70	518,00	3,60
2	WT	0	OP50+AA	490,00	999,00	280,00	493,00	1180,00	792,00	1270,00	1840,00	233,00	613,00	6,81	636,00	291,00	526,00	530,00	1360,00	5,41	465,00	129,00	24,60	494,00	1,02
3	WT	0	OP50+AA	556,00	1740,00	235,00	469,00	1140,00	756,00	1690,00	2520,00	53,30	656,00	19,50	891,00	605,00	497,00	850,00	1640,00	15,50	448,00	105,00	48,00	438,00	3,10
4	KO	0	OP50+AA	654,00	1230,00	270,00	569,00	1430,00	714,00	1480,00	2070,00	177,00	766,00	9,66	700,00	477,00	500,00	706,00	1950,00	7,64	459,00	124,00	30,80	429,00	1,65
5	KO	0	OP50+AA	435,00	1210,00	214,00	614,00	1380,00	785,00	1470,00	2130,00	135,00	845,00	12,10	865,00	483,00	525,00	780,00	1550,00	10,90	435,00	129,00	40,60	536,00	1,89
6	KO	0	OP50+AA	462,00	1640,00	233,00	424,00	1280,00	917,00	1160,00	1570,00	159,00	757,00	32,50	572,00	454,00	460,00	601,00	1890,00	8,41	462,00	114,00	26,60	451,00	7,50
7	-	0	OP50+AA	431,00	1300,00	251,00	417,00	867,00	766,00	1240,00	2030,00	158,00	853,00	6,35	886,00	531,00	411,00	680,00	1620,00	6,39	484,00	93,20	34,00	436,00	1,07
8	WT	0	H2O	3,62	27,10	1,06	1,71	0,67	0,75	0,83	2,60	0,48	1,70	1,85	2,94	0,69	0,88	1,73	2,80	0,06	0,49	0,11	0,13	1,85	0,52
9	KO	0	H2O	0,51	5,35	0,25	0,43	0,29	0,25	0,33	0,52	0,18	1,25	0,63	1,64	0,19	0,21	0,27	0,37	0,30	0,09	0,03	0,08	0,58	0,08
10	WT	60	OP50+AA	451,00	1580,00	25,70	579,00	1570,00	481,00	1490,00	2530,00	0,21	140,00	18,10	850,00	610,00	652,00	929,00	1390,00	16,20	456,00	62,70	56,20	497,00	3,74
11	WT	60	OP50+AA	798,00	2060,00	60,50	562,00	1840,00	582,00	1840,00	2920,00	0,38	198,00	23,50	1000,00	450,00	671,00	1040,00	1560,00	21,20	578,00	68,70	57,70	478,00	4,49
12	WT	60	OP50+AA	344,00	2010,00	38,70	621,00	1490,00	674,00	1750,00	2580,00	0,29	159,00	22,80	847,00	487,00	558,00	930,00	1720,00	13,50	479,00	75,70	53,00	503,00	4,63
13	KO	60	OP50+AA	429,00	2270,00	24,00	537,00	1420,00	663,00	1360,00	2240,00	0,26	49,70	20,50	534,00	522,00	486,00	780,00	1500,00	12,90	518,00	49,60	55,90	484,00	7,69
14	KO	60	OP50+AA	484,00	1850,00	19,40	499,00	1090,00	463,00	1670,00	2460,00	0,66	67,80	33,30	655,00	463,00	752,00	861,00	1780,00	10,20	456,00	70,20	44,40	551,00	9,97
15	KO	60	OP50+AA	435,00	2120,00	5,48	509,00	1510,00	345,00	1770,00	2700,00	0,31	52,90	28,00	678,00	514,00	586,00	891,00	1260,00	23,20	404,00	52,70	54,10	499,00	9,98
16	-	60	OP50+AA	459,00	1730,00	39,00	641,00	1310,00	555,00	1430,00	2340,00	0,30	313,00	13,20	1050,00	583,00	489,00	770,00	1540,00	14,50	337,00	59,60	49,10	469,00	1,23
17	WT	60	H2O	9,93	209,00	1,80	1,32	7,73	1,29	2,30	6,14	0,39	3,77	16,60	8,72	1,69	2,32	3,34	1,78	0,13	1,24	0,19	0,61	4,86	2,54
18	KO	60	H2O	1,48	108,00	0,62	0,79	2,05	0,50	0,49	3,52	0,12	1,85	7,81	2,45	0,11	1,24	0,68	0,66	0,18	0,14	0,09	0,90	3,17	2,54
19	WT	180	OP50+AA	693,00	1640,00	0,88	497,00	1220,00	2,37	1470,00	2380,00	0,21	77,10	23,40	471,00	582,00	621,00	842,00	1530,00	13,80	489,00	5,49	57,00	547,00	4,98
20	WT	180	OP50+AA	658,00	1520,00	6,06	567,00	1480,00	385,00	1600,00	2550,00	0,43	23,60	42,30	757,00	455,00	622,00	892,00	1510,00	13,80	580,00	44,10	61,30	528,00	5,17
21	WT	180	OP50+AA	554,00	1500,00	4,08	489,00	1140,00	215,00	1240,00	2140,00	0,72	52,50	34,60	532,00	625,00	745,00	824,00	1590,00	8,78	336,00	34,70	58,80	546,00	6,87
22	KO	180	OP50+AA	432,00	1660,00	0,38	578,00	1440,00	1,50	1740,00	2540,00	0,31	37,90	34,20	325,00	538,00	549,00	911,00	1760,00	14,70	383,00	1,38	48,70	462,00	9,03
23	KO	180	OP50+AA	575,00	1810,00	0,63	565,00	1310,00	1,52	1420,00	2040,00	0,31	42,20	32,70	245,00	522,00	677,00	845,00	1430,00	10,70	405,00	0,43	45,00	513,00	12,90
24	KO	180	OP50+AA	1243,33	2293,33	0,61	953,33	1406,67	0,87	1890,00	3083,33	1,05	77,67	57,33	473,33	586,67	680,00	1306,67	1706,67	19,80	480,00	0,42	67,33	650,00	13,37
25	-	180	OP50+AA	846,00	1570,00	0,55	498,00	1260,00	3,26	1600,00	2620,00	0,41	34,10	11,80	567,00	514,00	603,00	875,00	1340,00	26,60	405,00	10,20	50,60	451,00	1,18
26	WT	180	H2O	22,60	294,00	0,95	0,25	4,12	0,38	1,24	6,00	0,37	2,36	21,40	2,88	0,15	1,74	2,02	0,25	0,21	0,18	0,09	1,18	4,34	3,34
27	KO	180	H2O	2,76	135,00	0,16	0,25	3,34	0,12	0,16	0,50	0,15	0,60	8,67	0,88	0,01	2,00	0,18	0,03	0,06	0,03	0,02	1,48	4,48	2,98
28	WT	360	OP50+AA	696,00	1660,00	0,73	267,00	1790,00	2,42	1830,00	2580,00	0,48	66,90	18,20	2,95	655,00	618,00	879,00	1570,00	13,70	427,00	1,86	48,30	510,00	8,38
29	WT	360	OP50+AA	647,00	2300,00	4,06	634,00	1790,00	28,30	1770,00	2840,00	1,45	40,90	53,20	684,00	557,00	705,00	953,00	1680,00	20,90	503,00	35,00	57,00	526,00	12,70
30	WT	360	OP50+AA	976,00	1830,00	2,36	599,00	1600,00	4,82	1420,00	2300,00	1,15	29,10	52,60	675,00	552,00	743,00	935,00	1510,00	10,30	519,00	3,77	56,30	560,00	10,80
31	KO	360	OP50+AA	516,00	1240,00	2,38	167,00	1880,00	3,50	1670,00	2190,00	0,82	76,00	15,00	19,50	427,00	660,00	753,00	1340,00	12,90	341,00	2,33	66,30	601,00	15,50
32	KO	360	OP50+AA	253,00	1070,00	0,92	34,60	1430,00	1,14	1490,00	2180,00	0,72	58,90	26,50	3,44	465,00	732,00	681,00	1340,00	13,60	353,00	1,77	53,80	507,00	19,50
33	KO	360	OP50+AA	25,30	809,00	0,52	3,48	1920,00	1,08	1770,00	2820,00	1,04	112,00	12,80	3,09	462,00	742,00	727,00	1220,00	22,40	380,00	1,87	70,20	634,00	18,50
34	-	360	OP50+AA	510,00	1180,00	0,88	261,00	1370,00	1,72	1660,00	2560,00	0,28	36,00	14,00	30,00	586,00	681,00	1040,00	1570,00	25,00	444,00	4,39	63,70	537,00	2,05
35	WT	360	H2O	3,73	121,00	1,23	0,70	1,40	1,13	1,11	2,50	0,62	2,15	10,40	7,08	0,55	5,98	1,96	7,97	0,12	0,94	0,33	1,14	12,50	5,62
36	KO	360	H2O	1,80	108,00	0,15	0,13	0,44	0,10	0,07	0,54	0,20	1,14	9,11	2,07	0,02	1,19	0,30	0,10	0,03	0,06	0,02	1,61	3,35	2,33

original data from Analyst ® 1.5 Software (AB Sciex, Darmstadt, Germany)

Sample	Protein content [g/l]
01 - WT 1 C. elegans, AA, OP50	6,03
02 - WT 2 C. elegans, AA, OP50	5,89
03 - WT 3 C. elegans, AA, OP50	6,22
04 - pept-1 4 C. elegans, AA, OP50	2,7
05 - pept-1 5 C. elegans, AA, OP50	3,94
06 - pept-1 6 C. elegans, AA, OP50	4,92
08 - WT C.elegans, water	6,23
09 - pept-1 C.elegans, water	3,34
