# Supplementary material for: The Reproduction Rate of Peptide Transporter PEPT-1 Deficient C. elegans Is Dependent on Dietary Glutamate Supply
Source: Front Mol Biosci. 2018 Nov 30;5:109. doi: 10.3389/fmolb.2018.00109 (PMC6284198; doi:10.3389/fmolb.2018.00109)
Supplement: Supplementary file 2 [file Image_1.pdf]

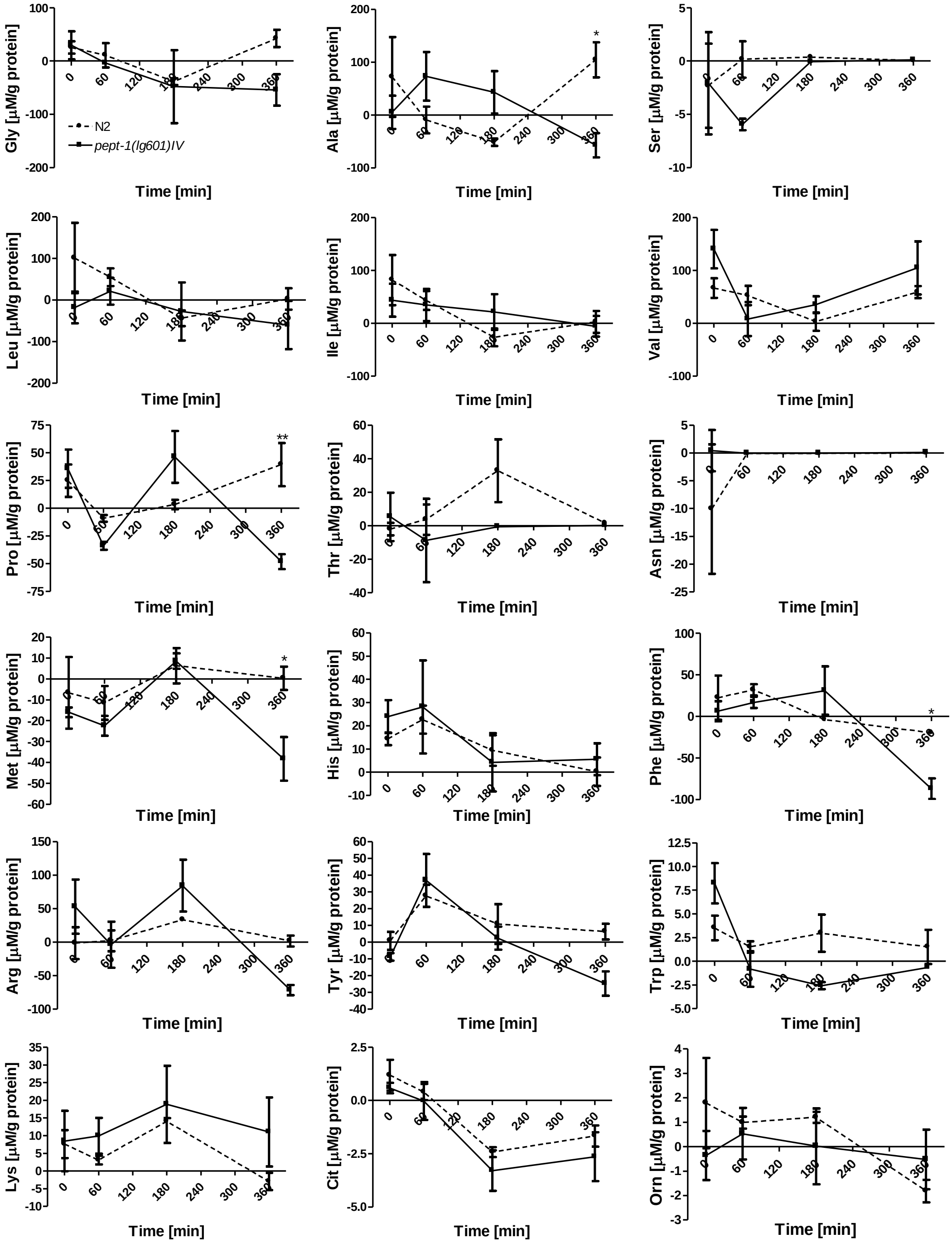

Supplemental figure S1: Overview of the concentration changes of individual amino acids over time in culture medium of wildtype (N2) and *pept-1(lg601) C. elegans*: Medium samples were analyzed after 0 min, 60 min, 180 min and 360 min. Values are presented as mean ± SEM (n = 3 biological replicates; *: P < 0.05, **: P < 0.01; mixed model two-way ANOVA with Bonferroni post test).
